# P-2120. Toxicity and Adverse Outcomes of Letermovir vs. Valganciclovir Cytomegalovirus (CMV) Prophylaxis in Lung Transplantation

**DOI:** 10.1093/ofid/ofaf695.2284

**Published:** 2026-01-11

**Authors:** Dimitrios G Moshovitis, Andres Franceschi Coll, Ahmad Nawlo, Marco Aurelio Diaz, Christine Atallah, Paul Sakr, Hassan Alshaker, Rayven Frierson, Djenabou Sow, Jawad Safiia, Pritha Sen, Dimitrios Farmakiotis, Sophia Koo

**Affiliations:** Brigham and Women's Hospital, Boston, MA; Brigham and Women’s Hospital, Boston, Massachusetts; Brigham and Women's Hospital, Boston, MA; Brigham and Women's Hospital, Boston, MA; Brigham and Women's Hospital, Boston, MA; Brigham and Women's Hospital, Boston, MA; Brigham and Women's hospital, Boston, Massachusetts; Brigham and Women's Hospital, Boston, MA; Brigham & Women's Hospital, Boston, Massachusetts; Brigham and Women’s Hospital, Boston, Massachusetts; Brigham and Women's Hospital, Boston, MA; Beth-Israel Deaconess Medical Center; Brigham and Women's Hospital, Dana-Farber Cancer Institute, Boston, MA

## Abstract

**Background:**

CMV remains a significant cause of morbidity and mortality in lung transplant (LT) recipients. While valganciclovir (vGCV) has been a mainstay of CMV prophylaxis, it is associated with treatment-limiting side effects, such as myelosuppression. Letermovir (LET), an inhibitor of the CMV DNA terminase complex, is a potential alternative.

**Table 1:**
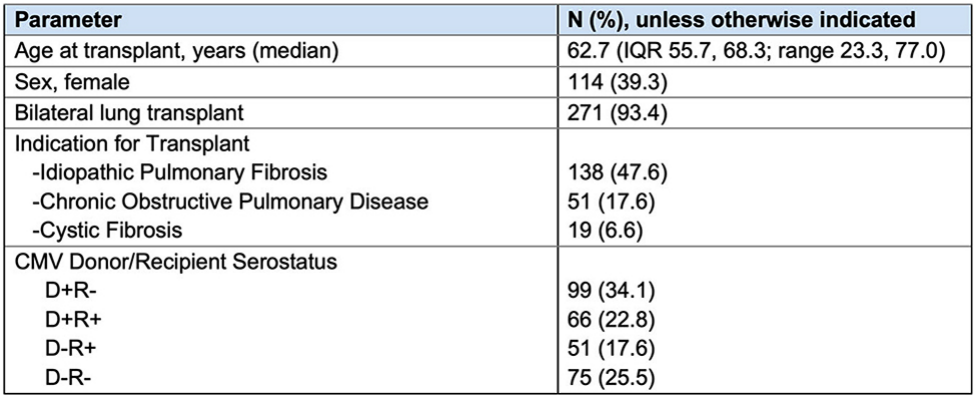
Patient characteristics

**Table 2:**
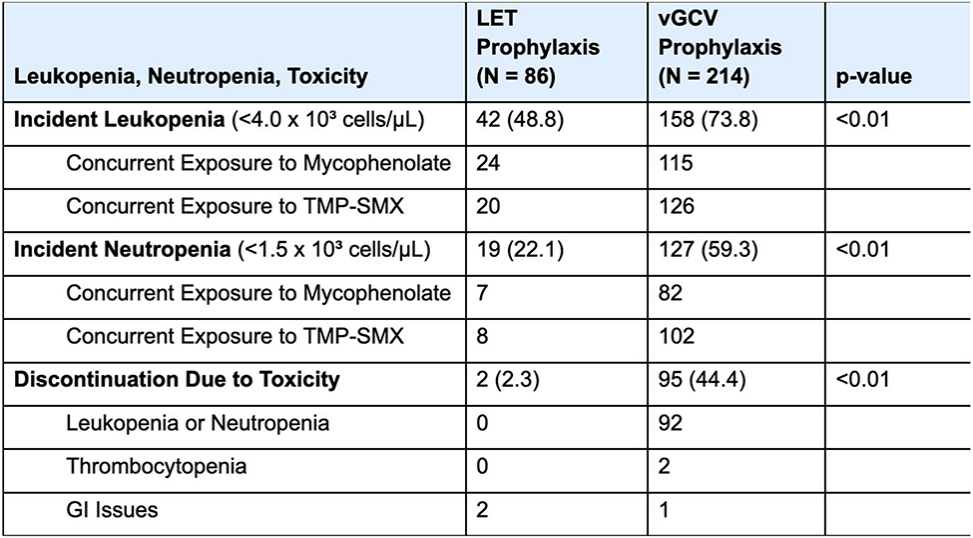
Leukopenia, neutropenia, and toxicity events on LET or vGCV

**Methods:**

We conducted a retrospective cohort study in 290 adult LT recipients at a large academic medical center from 1/2017-9/2022, examining toxicities, adverse outcomes, and CMV reactivation rates associated with LET vs. vGCV prophylaxis. We used Fisher’s exact tests to compare toxicity rates between these groups. We used Cox models to examine the association between LET and vGCV as time-varying exposures with incident leukopenia and neutropenia.

**Table 3:**
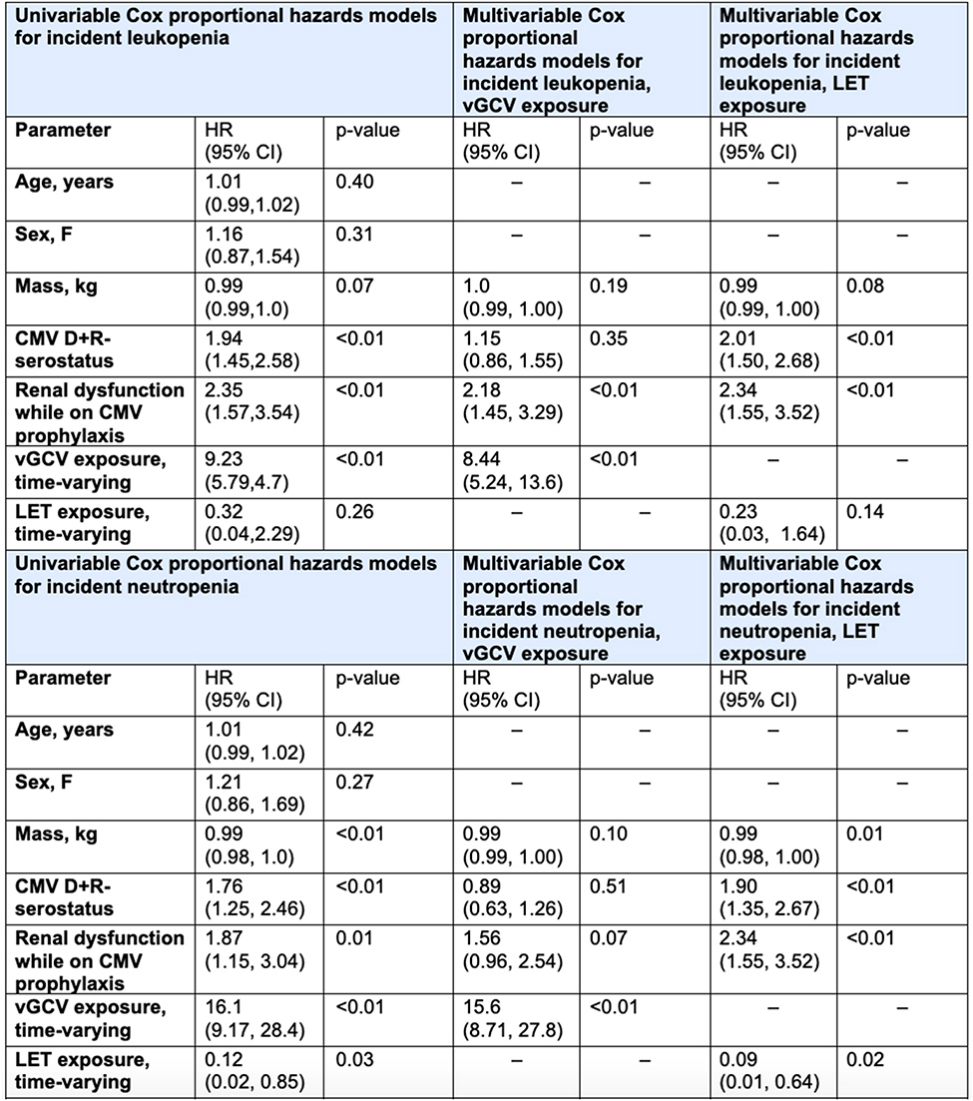
Cox proportional hazards models for incident leukopenia (WBC < 4.0 x 10³ cells/μL) and neutropenia (ANC < 1.5 x 10³ cells/μL)

**Figure:**
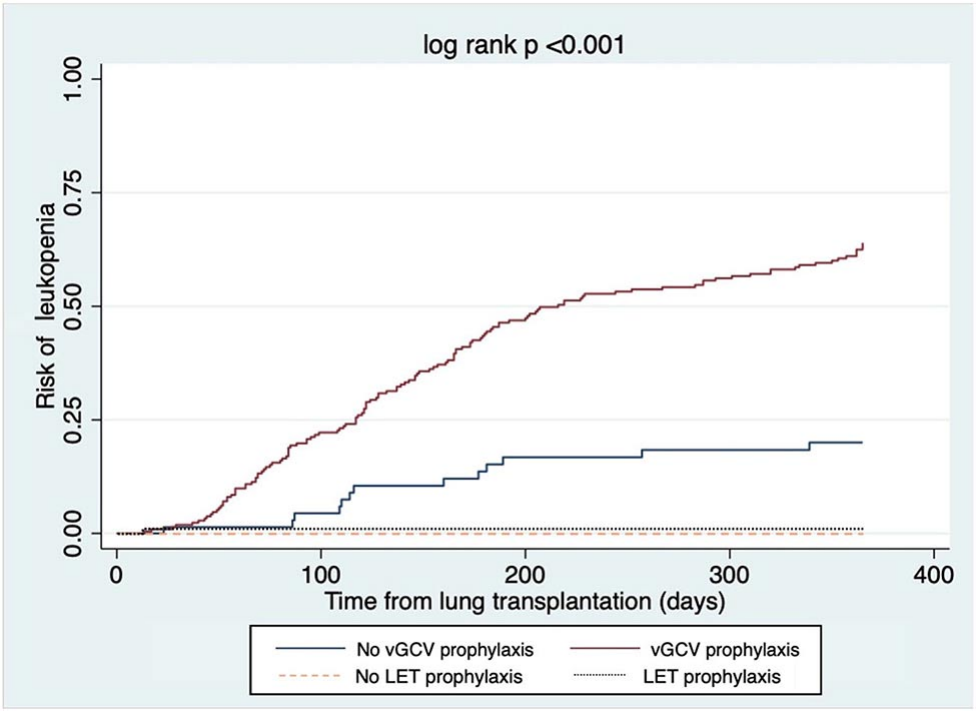
Kaplan-Meier plot of leukopenia risk in patients receiving vGCV prophylaxis or LET prophylaxis

**Results:**

Of 290 LT recipients (Table 1), 86 (29.7%) received LET prophylaxis for a median 281 (IQR 171, 442) days, and 214 received vGCV prophylaxis for 332 (IQR 164, 518) days. Of 214 who received vGCV, 158 (73.8%) developed leukopenia and 127 developed neutropenia during vGCV exposure (Table 2, Figure). Of 86 patients who received LET, 42 (48.8%) developed leukopenia and 19 (22%) developed neutropenia during LET exposure. Rates of leukopenia, neutropenia, and discontinuation due to CMV prophylaxis toxicity were all significantly higher in patients who received vGCV prophylaxis, compared to those who received LET. With LET or vGCV exposure modelled as time-varying covariates, CMV D+R- serostatus, renal dysfunction while receiving CMV prophylaxis, and vGCV exposure were associated with an increased risk of leukopenia (Table 3). In multivariable models, renal dysfunction on CMV prophylaxis and vGCV exposure were associated with leukopenia, adjusting for other covariates, while LET was not associated with an increased risk of leukopenia. One patient had CMV reactivation requiring an antiviral change on LET 8 days after starting LET, and 15 patients (7.0%) had CMV reactivation while receiving vGCV a median of 450 (IQR 204, 607) days after starting vGCV.

**Conclusion:**

LET prophylaxis was associated with a lower rate of toxicity than vGCV in LT recipients, without higher rates of CMV developing on treatment. vGCV exposure represented a significant risk for developing leukopenia, while LET exposure did not.

**Disclosures:**

Sophia Koo, MD, SM, Ansun BioPharma: Grant/Research Support|Generate Biomedicines: Advisor/Consultant|GlaxoSmithKline: Grant/Research Support|Locus Biosciences: Grant/Research Support|Merck Sharp & Dohme: Grant/Research Support|Scynexis, Inc: Grant/Research Support|Vertex Pharmaceuticals: Advisor/Consultant

